# Health-related quality of life and determinants in North-China urban community residents

**DOI:** 10.1186/s12955-020-01522-w

**Published:** 2020-08-14

**Authors:** Hui Wu, Shengbo Han, Guicheng Zhang, Weidong Wu, Naijun Tang

**Affiliations:** 1 School of Public Health, Xinxiang Medical University, 22 Qixiangtai Rd, Tianjian, 300070 China; 2 School of Public Health, Tianjin Medical University, 601 Jinsui Rd, Xinxiang, 453000 Henan China; 3 Zhengzhou Traditional Chinese Medicine Hospital, 65 wenhuagong Rd, Zhongyuan District, Zhengzhou, 450002 Henan China; 4 School of Public Health, Curtin University, Roberts Rd, Subiaco WA, Perth, 6008 Australia

**Keywords:** Chinese urban communities, Quality of life, Chronic diseases, SF-36

## Abstract

**Background:**

The objectives of this study were to investigate the HRQoL of residents living in central urban areas (CUA) and developing neighborhoods (DN) areas of North-China and to examine the relationship between health conditions and the physical and mental components of quality of life.

**Methods:**

A stratified random sample was taken and health survey scoring system questionnaire SF-36 was used to conduct the HRQoL survey among community residents in the two selected districts in 10 cities. A general questionnaire was also administered with questions that collected general information, population demographic characteristics and health behaviours, social relationships and perception of life satisfaction.

**Results:**

Five thousand eight hundred eighty-one questionnaires were returned from 6059 invitations with a effective response rate of 97%. The residents in DN had a higher score of physical function, role limitation due to physical problems and vitality than those living in CUA. The prevalence of several chronic diseases was lower in DN’s residents than CUA’s residents. Age, presence/absence of chronic diseases, leisure time exercise, regular daily routine, sleep quality, appetite, family and social relationships and life satisfaction were significant determinants of HRQoL.

**Conclusions:**

Residents living in newly developed neighborhoods in China while keeping some habits and lifestyles of their original rural communities are healthier in terms of chronic diseases and HRQoL. Together with other risk factors chronic diseases are an important determinant on HRQoL. Several healthy habits and behaviors such as having a regular daily routine and exercising during leisure time improved HRQoL in Chinese urban communities. Targeted policies of public health based on these findings can better the health-related quality of life.

## Introduction

Health-related quality of life (HRQoL) is a multifaceted concept that includes social functioning and physical and mental health status. It is an interesting and important research topic as the findings of such research provide fundamental knowledge and evidence for policy makers and government administration to implement targeted policy intervention and address significant health issues that are related to people’s health status and functional wellbeing. The recent rapid change in Chinese society affects a large population of about 1.4 billion, approximately one fifth of the world population. It is timely to assess HRQoL in Chinese communities, particularly in the context of rapid urbanisation [1]. These studies will provide data for implementing evidence-based interventions in health care and public health by relevant health organisations. Supplementary to studies conducted in developed countries, HRQoL studies in China will add valuable data from the perspective of developing countries.

During the past 30 or 40 years, standard of living, health care and public health have significantly improved as part of the economic reform in China. However, Chinese communities are also confronting new challenges in promoting physical and mental wellbeing and reducing health inequalities. Rapid urbanization and the expansion of the urban population, an increasingly aging population, increased prevalence of non-communicable chronic diseases, and severe air pollution due to early industrialization as well as internal migration have introduced new issues into Chinese communities that may adversely influence HRQoL for residents [2–4]. These concerns are a key priority in public health, however to date, only a few studies on HRQoL have been conducted in Chinese communities [5–7].

The MOS 36-item short form health survey (SF-36) has been used world-wide as a generic and coherent measure to assess HRQoL in patients and residents [8–10]. More than 40 translated versions have been applied around the world [11] and it is the standard for international quality of life assessment. The Chinese version of SF-36 was first developed and tested in American Chinese and Cantonese [12]. In 2003 a Chinese version of SF-36 for mainland Chinese was developed and validated by Li et al. [13], and since then it has been commonly used in Chinese communities. In the present study we used the Chinese version (mainland) of SF-36 to compare HRQoL of residents living in an older urban city central area with residents living in a newly developed urban area. The new urban area was recently transformed from a rural suburb. We also investigated which determinants and factors are associated with HRQoL in these communities.

## Methods

### Study design

This cross-sectional study was conducted in North-China to evaluate HRQoL in urban area which including Zhengzhou, Xinxiang, Luoyang, Nanyang, Shijiazhuang, Handan, Qingdao, Binzhou and Jining, 10 cities in all. A sample size of 700 was determined sufficient in each of the survey cities, using the formula n = (u_α_σ/δ)^2^ (α: Type I error, u_α:_ standard normal distribution, when α =0.05, u_α_ = 1.96) with a two-sided significance of 0.05, a HRQoL scoring error of less than 2.5 points (δ), a standard deviation σ = 30 and a response rate of 80%. The household was chosen as the sampling unit, using a stratified random sampling method. Taking Zhengzhou as an example, the sample is as follows: The Jinshui district was randomly selected as the central urban area (CUA) and the Huiji district was randomly selected as the developing neighborhood (DN). Jinshui has a population of approximately 1.6 million and Huiji, a population of about three hundred thousand. We sampled two neighborhoods at random in each district, and sampled 30 ordinary households at random in each neighborhood. Thus a total of 120 families were included in this study.

The Chinese version of SF-36 V2, which was translated and validated by Professor Li Lu of Zhejiang University, was used for the present study [13]. In all, we investigated a total of 1200 families consisting of 6059 residents across both CUA and DN. A total of 5982 questionnaires were administered. One hundred one questionnaires were excluded due to incomplete information and 5881 questionnaires were used for further analysis.

All participants were sent a letter explaining the aim of the study, who could take part in after give consent. All respondents were told that the results of this investigation would be used for research purposes only and that all personal information was confidential. Ethics approval was obtained from the Xinxiang Medical University ethics committee and the survey was recorded from April of 2016 to January of 2018. All participants enrolled gave their written informed consent.

### Participants

To be included the participants had to be aged 14 years or over, not suffering from dementia or any other deurodegenerative diseases and competent of completing the self-report questionnaires without assistance.

### Data collection

This study used face-to-face questionnaire. Sociodemographic characteristics of the participants (age, gender, marital status, educational level, occupation), income, medical insurance, health related lifestyle (smoking, drinking, exercise, daily routine, appetite, sleeping), their current health status according to the ICD-10 classification,relational problem(s) with friends or relatives were self-reported by participants and recorded.

HRQoL was assessed by the Chinese version of SF-36 V2 questionnaire, which are divided into eight dimensions: Physical Function (PF), Role limitations due to Physical problems (RP), Bodily Pain (BP), General Health (GH), Vitality (VT), Social Function (SF), Role limitations due to Emotional problems (RE) and Mental Health (MH). The first four dimensions are defined as Physical Health Components (PHC) while the last four dimensions are defined as Mental Health Components (MHC).

### Statistical analyses

The data from the survey was logged using Epidata 3.1 and statistical analysis was performed using SPSS version 20.0 software (SPSS Inc., Chicago, IL, USA). The comparison for categorical and continuous variables between CUA and DN was performed using Chi-square tests and independent samples T tests, respectively. We followed the recommendations of Wagner AK et al. [14] for our scoring calculations on HRQoL. To compare the scores of the 8 dimensions and 2 summary components of SF-36 between residents living in CUA and DN a general linear model was used adjusted for age and gender. These HRQoL scores were also associated with presence/absence of common chronic diseases after adjusting for age, gender and the residents’ areas. After adjusting for the number of common chronic diseases and other confounders the variables for demographic characteristics and health behaviours, social relationships and perception of life satisfaction were correlated with PHC and MHC (of SF-36) in a general linear model. The final stepwise regression analysis for PHC and MHC included variables of age, gender, residents’ areas, number of chronic diseases and the significant variables of demographic characteristics and health behaviours, social relationships and perception of life. For all tests α = 0.05 was chosen as the level for statistical significance.

## Results

There were 3105 participants (52.8%) from CUA and 2776 (47.2%) from DN. Table 1 shows the population characteristics of the participants stratified by the two areas. Compared with CUA, DN had a lower percentage of participants with tertiary or higher education, a lower percentage of participants with higher income, a lower percentage of employed and retired participants and a higher percentage of participants that are un-employed or housekeepers.

**Table 1:** The selected population characteristics of the community residents living in a central city area (CUA) and a developing neighbourhood (DN)

	CUA (*n* = 3105)	DN (*n* = 2776)	*P*
Age, years	48.8 ± 12.6	49.3 ± 13.1	0.136
Females: n (%)	1847 (59.5)	1693(61.0)	0.240
Education level, n (%)			<0.001
Tertiary or higher	975 (31.4)	264(9.5)	
Secondary or high school	1832 (59.0)	1810 (65.2)	
Primary or lower	298 (9.6)	702(25.3)	
Marriage status, n (%)			0.292
Single	624 (20.1)	591 (21.3)	
Married or cohabiting	2195 (70.7)	1954 (70.4)	
Divorced or widow/widower	286 (9.2)	230 (8.3)	
Employment status, n (%)			<0.001
Student	171 (5.5)	144 (5.2)	
Un-employed or housekeeper	807 (26.0)	1108 (39.9)	
Employed	1267 (40.8)	994 (35.8)	
Retired	860 (27.7)	530 (19.1)	
Income, n (%)			<0.001
Low	730 (23.5)	836 (30.1)	
Middle	1882 (60.6)	1549 (55.8)	
High	494 (15.9)	391 (14.1)	
Insurance, n (%)			
No	180 (5.8)	169 (6.1)	0.638
Yes	2925 (94.2)	2607 (93.9)	
Smoking, n (%)			0.109
No	2270 (73.1)	2088 (75.2)	
Sometimes	317 (10.2)	244 (8.8)	
Everyday	519 (17.7)	444 (16.0)	
Drinking, n (%)			0.290
No	2192 (70.6)	2007 (72.3)	
Sometimes	543 (17.5)	469 (16.9)	
Often	370 (11.9)	300 (10.8)	
Chronic disease, n (%)			0.027
No	1860 (59.9)	1741 (62.7)	
Yes	1245 (40.1)	1035 (37.3)	

### Prevalence of chronic diseases and health-related quality of life in CUA and DN

Ten common chronic diseases were investigated: hypertension, intervertebral disc related diseases, arthritis, heart disease, gastroenteritis, cerebrovascular disease, chronic obstructive pulmonary disease (COPD)/asthma, diabetes mellitus, cholecystitis and cholelithiasis and mental illness. Hypertension (23.3%) and diabetes mellitus (10.8%) were the highest crude prevalence of these 10 chronic diseases. The prevalence of hypertension, heart disease and asthma/COPD was significantly lower in DN than CUA (19.5% vs 27.2%, *P*<0.001; 4.8 vs. 9.6%, *P*<0.001; 3.7% vs 5.4%, *P* = 0.002, respectively).

DN residents had a higher mean score for 3 of 8 dimensions of the HRQoL questionnaire, adjusted for age and gender (Table 2). The mean scores of PF, RP and VT were significantly higher in residents living in DN than those in CUA. There was no significant difference between the two areas for the Physical and Mental Health Components.

**Table 2:** Adjusted mean scores of SF-36 of urban community residents living in a central urban area (CUA) and a developing neighbourhood (DN)

	CUA (*n* = 3105)			DN (*n* = 2776)			
		95% CI			95% CI		
	Mean	Lower	Upper	Mean	Lower	Upper	*P*
PF	81.1	78.1	84.1	86.0	83.0	89.0	0.023
RP	80.9	78.6	83.2	85.1	82.7	87.4	0.013
BP	89.2	86.6	91.8	90.0	87.4	92.6	0.67
GH	65.3	63.5	67.1	66.1	64.4	67.9	0.52
VT	44.2	42.8	45.6	47.8	46.4	49.3	< 0.001
SF	53.7	52.4	55.0	53.7	52.4	55.0	0.98
RE	84.7	82.6	86.8	85.5	83.3	87.6	0.62
MH	60.6	59.2	62.1	61.6	60.2	63.1	0.35
PHC	321.0	314.1	327.8	328.6	321.8	335.5	0.12
MHC	243.3	239.4	247.2	248.6	244.8	252.5	0.055

Age and gender were adjusted for and general linear model was employed for the analysis

### Chronic diseases and health-related quality of life scores

After adjusting for confounding effects of age, gender and residents’ area, we investigated the associations of presence/absence of the common chronic diseases with HRQoL scores. Mental illness was excluded due to the small number of residents (*n* = 30) who self-reported this. Table 3 shows adjusted mean scores of HRQoL in these residents with and without common chronic diseases. Residents with chronic conditions mostly had a lower quality of life scores, relative to residents without the condition, particularly for physical component domains such as PF, RP, BP and GH. Hypertension was with a more than 10% decrease in scores of RP, BP, GH and PHC, intervertebral disc related disease in scores of BP, GH and PHC, arthritis in scores of RP, BP, GH and PHC, heart disease in scores of PF, RP, BP, GH, PHC, gastroenteritis in scores of BP, GH, MH and PHC, cerebrovascular disease in scores of PF, RP, BP, GH, RE, MH and PHC, COPD/Asthma in scores of PF, RP, BP, RE and PHC, diabetes mellitus in scores of PF, RP, BP, GH, RE and PHC and cholecystitis and cholelithiasis in the score of GH.

**Table 3:** Adjusted mean scores of SF-36 for urban community residents with and without common chronic diseases

	Hypertension		Diabetes mellitus		Gastroenteritis		Heart disease		Intervertebral disc related diseases		Cholecystitis and Cholelithiasis		Cerebrovascular disease		Arthritis		COPD/Asthma	
	No	Yes	No	Yes	No	Yes	No	Yes	No	Yes	No	Yes	No	Yes	No	Yes	No	Yes
PF	84.1	79.9	84.3	62.5***	84	77.9	85.4	61.3***	84	80	83.7	78.5	85.1	58.8***	84.1	77.1	84.4	64.4***
RP	85	72.9***	83.4	70.6**	83.5	76.0*	84.2	68.0***	83.7	76.8*	83	81.6	84.3	62.2***	83.9	72.0***	83.5	72.0**
BP	91	81.2**	90.2	74.2**	90.5	78.5**	91.1	71.5***	91.3	74.5***	90	84.3	90.6	73.8***	91.4	68.2***	90.2	76.2**
GH	67.1	57.1***	66	56.4**	66.3	58.6**	66.6	54.5***	66.8	55.9***	66	54.2**	66.5	53.2***	66.5	55.8***	66	60.5
VT	45.8	47.8	46.1	43.4	46	45.8	45.4	53.3***	45.9	47.5	46	48.8	45.9	48.5	46.2	44	45.9	49.8
SF	53.6	54.8	53.6	56.3	53.7	53.6	53.7	54.8	53.7	54.3	53.7	53.3	53.6	56.3	53.6	54.8	53.8	52.5
RE	85.5	82.2	85.5	71.6**	85.6	78.4*	85.6	78.3*	85	85.4	85.3	78.2	86.2	67.7***	85.4	81	85.5	74.8**
MH	61.7	57.6*	61.2	59.2	61.7	54.0***	61.5	56.6*	61.3	59.3	61.2	57.2	61.8	50.9***	61.5	57.1*	61.3	57.4
PHC	329.7	293.1***	326.7	270.3***	327.2	292.6***	329.3	268.7***	328.8	288.7***	325.4	303.6	328.1	255.1***	329	274.0***	326.7	283.5**
MHC	246.5	242.4	246.5	230.6*	247	231.9**	246.2	242.9	245.9	246.5	246.2	237.5	247.4	223.4***	246.7	236.8	246.5	234.5

General linear model was employed for the analysis with age, gender and areas (CUA and DN) adjusted for. *: *P* < 0.05; **: *P* < 0.01; ***: *P* < 0.001

Table 4 shows the adjusted mean scores of SF-36 as function of the number of common chronic diseases that residents have. There is an inverse association between the number of chronic diseases and nearly all domains of the SF-36 with a linear significance of *P* < 0.001, except for the VT and SF domains.

**Table 4:** Adjusted mean scores of SF-36 versus the number of chronic diseases reported by urban community residents

	Number of chronic diseases				
	0	1	2–3	> 3	*P*__ linear_
PF	86.7	83.3	76.8*	46.3***	< 0.001
RP	87.7	79.2***	70.7***	57.2***	< 0.001
BP	97.3	81.1***	69.1***	66.0***	< 0.001
GH	70.6	60.9***	52.7***	46.5***	< 0.001
VT	45.8	44.8	48.1	52.1*	0.11
SF	53.6	53.2	54.6	56.4	0.44
RE	87.9	82.8**	77.4***	71.6***	< 0.001
MH	63.7	57.8***	55.2***	54.0**	< 0.001
PHC	345.1	306.9***	271.1***	228.5***	< 0.001
MHC	251.0	238.6***	235.3**	234.1*	< 0.001

Adjusted for age, gender and area. General linear model was employed for the analysis and the mean scores in residents with 1, 2–3, and > 3 chronic diseases were compared to those in residents without chronic conditions: *: *P* < 0.05; **: *P* < 0.01; ***: *P* < 0.001

### Demographic characteristics and health-related quality of life scores

We examined the association of social demographic characteristics with the summary HRQoL scores PHC and MHC. Education level, marriage status, employment, income and medical insurance were investigated using a general linear model before and after adjusting for the number of chronic diseases. Table 5 shows the statistical significance of these six social demographic variables. Marriage status and employment were significantly associated with PHC scores after adjusting for age, gender and residents’ area. However, after adjusting for the number of chronic diseases the significance did not remain.

**Table 5:** Statistical significance for associations of social demographic characteristics with the scores of physical health component (PHC) and mental health component (MHC) assessed by SF-36

	PHC		MHC	
	p1	p2	p1	p2
Education	0.21	0.21	0.98	0.98
Marriage#	0.032	0.09	0.076	0.11
Employment#	0.001	0.067	0.87	0.75
Income	0.38	0.071	0.073	0.063
Insurance	0.27	0.42	0.14	0.17

General linear model was used for the analysis. p1: p values after adjusting for age, gender and areas (CUA and DN); p2: *p* values after adjusting for the confounders for p1 plus number of chronic disease. #: 315 students were excluded in the analysis

### Obesity, health behaviours, social relationships and perception of life satisfaction with health-related quality of life scores

**Fig. 1:**
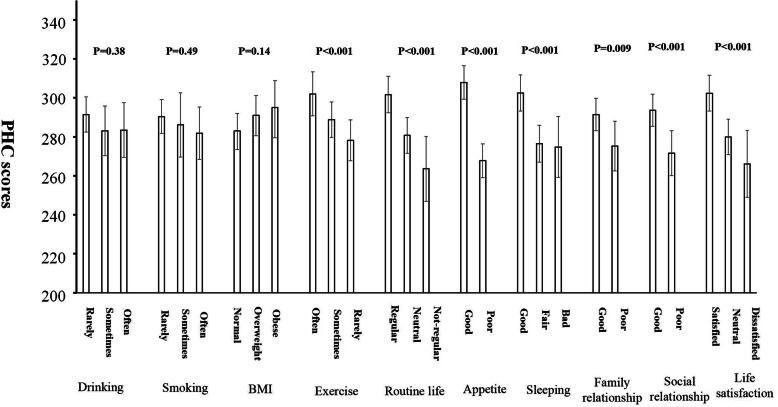
Adjusted mean scores of PHC (adjusting for age, gender, residents’ areas and the number of chronic diseases)

**Fig. 2:**
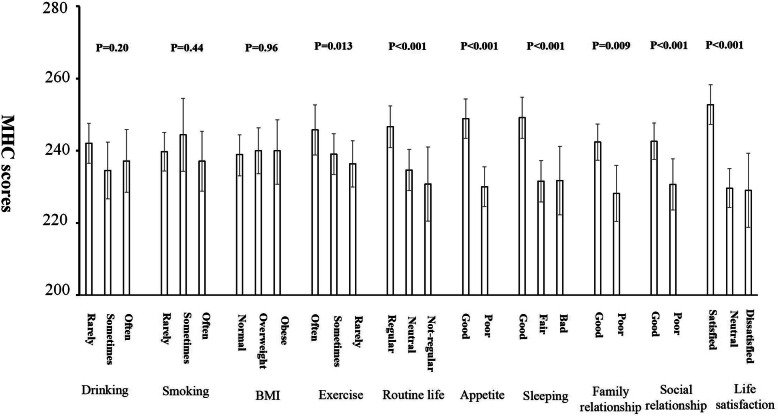
Adjusted mean scores of MHC (adjusting for age, gender, residents’ areas and the number of chronic diseases)

We also investigated the association of obesity, health behaviours, social relationships and perception of life satisfaction with the HRQoL scores of PHC and MHC. Table 6 shows the statistical significance of the 10 relevant variables: obesity, smoking, drinking, exercise, daily routine, appetite, sleeping, family relationship, social friendship and life satisfaction. Obesity and smoking and drinking behaviour were not associated with HRQoL in this population. However, leisure time exercise, daily routine habit, good appetite and sleeping quality, good family and social relationships and higher life satisfaction were strongly and positively correlated with PHC and MHC, independent of chronic diseases. Figures 1 and 2 show adjusted mean scores of PHC and MHC, respectively, after adjusting for age, gender, residents’ areas and the number of chronic diseases.

**Table 6:** Statistical significance for associations of obesity, health behaviours, social relationships and perception of life satisfaction with the scores of physical health component (PHC) and mental health component (MHC) assessed by SF-36

	PHC		NHC	
	P1	P2	P1	P2
Obesity	0.81	0.14	0.98	0.96
Smoking	0.12	0.48	0.32	0.43
Drinking	0.24	0.46	0.21	0.28
Exercise	< 0.001	0.001	0.016	0.038
Daily routine	< 0.001	< 0.001	< 0.001	< 0.001
Appetite	< 0.001	< 0.001	< 0.001	< 0.001
Sleeping	< 0.001	< 0.001	< 0.001	< 0.001
Family relationship	0.001	0.009	< 0.001	< 0.001
Friendship	< 0.001	< 0.001	< 0.001	0.001
Life satisfaction	< 0.001	< 0.001	< 0.001	< 0.001

General linear model was used for the analysis. p1: p values after adjusting for age, gender and areas (CUA and DN); p2: *p* values after adjusting for the confounders for p1 plus number of chronic disease

### Final models of stepwise regression of PHC and MHC

Age, gender, residents’ area, number of chronic diseases and significant variables of health behaviours, social relationships and perception of life satisfaction were further examined for the association with PHC and MHC using stepwise regression (Table 7). For PHC the final model includes age (*P* < 0.001), number of chronic diseases (*P* < 0.001), good appetite (*P* < 0.001), regular daily routine (*P* = 0.009), leisure exercise (*P* = 0.036) and life satisfaction (*P* = 0.012). These significant variables explained 34% of the PHC variations. For MHC the final model includes age (*P* = 0.044), sleeping quality (*P* = 0.003), appetite (*P* = 0.002 and life satisfaction (*P* < 0.001). The final model explained 14% of MHC variations.

**Table 7:** Final regression model of PHC and MHC

Determinants	Unit/coding	Beta	Standard error	*P*	Adjusted R square
**PHC**					0.34
Age	Years	−0.50	0.14	< 0.001	
Number of Chronic diseases	0,1,2,3	−30.6	3.0	< 0.001	
Appetite	0,1	31.6	4.7	< 0.001	
Regular daily routine	0,1,2	9.6	3.7	0.009	
Leisure time exercise	0,1,2	6.3	3.0	0.036	
Life satisfaction	0,1,2	9.1	3.6	0.012	
**MHC**					0.14
Age	Years	0.15	0.07	0.044	
Sleeping quality	0,1,2	6.9	2.3	0.003	
Appetite	0,1,2	9.9	3.1	0.002	
Life satisfaction	0,1,2	13.8	2.2	< 0.001	

## Discussion

Our study found that the newly developed neighbourhoods in the 10 cities still exhibit some features (Table 1) of their origins of rural community and have a lower prevalence of several common chronic diseases such as hypertension, heart diseases and COPD/asthma compared with the old central city areas. The HRQoL was generally comparable between the city central district and the newly developed neighbourhoods with slightly higher scores of physical function, role limitations due to physical problems and vitality in newly developed neighbourhoods’ residents, consistent with the lower prevalences of chronic diseases in the district. According to our findings public health strategies to improve HRQoL should be developed to promote the relatively healthy environments and lifestyles in the newly developed neighbourhoods.

Considering the two investigated districts as a whole, more than one in three residents had at least one common chronic disease. This high prevalence in urban residents is consistent with the trend of a global increase of chronic diseases [15, 16]. Slightly lower prevalence of chronic diseases in the newly developed neighbourhoods is considered due to the preservation of previous rural suburb environments and lifestyles. The risk is that without appropriate prevention strategies the prevalence of chronic diseases in newly developed neighbourhoods is likely to increase to the levels observed in the city central areas. It is likely that the urbanisation and industrialization in China may bring about a significant increase of incidence of chronic diseases in Chinese communities [16]. Therefore appropriate prevention and management strategies for chronic diseases should be implemented in these Chinese urban communities and corresponding allocations of public health resources should be considered.

The HRQoL of persons with chronic diseases was generally poorer than for persons without chronic diseases and patients affected by more than one chronic disease had further reduced HRQoL scores, particularly for physiological health. Consistent with our results recent studies also reported significant influences of chronic diseases on HRQoL in Chinese community residents [17–19]. All 9 common chronic diseases analysed in the present study were associated with significantly reduced scores of some domains assessed by SF-36 with an up to 31% in reduction of physical function (PF) for cerebrovascular disease. In our final stepwise regression model the presence of one more chronic diseases was accompanied with a reduction of 30.6 units in PHC scores. The linear correlation between the number of common chronic diseases and the HRQoL domains were all significant (*p* < 0.001) except for vitality (VT) and social function (SF). Our investigation has shown strong associations between chronic diseases and poor HRQoL. Therefore prevention and management of chronic diseases should be a key priority in community health in order to maintain a high level of HRQoL for Chinese urban communities.

Obesity is an important risk factor for many chronic diseases such as hypertension, coronary heart disease and diabetes. Studies have shown that obese persons have a reduced HRQoL compared to those with normal body weight [20, 21] although this is not always consistent [22]. In our study obesity was not associated with HRQoL. To explain the absent relationship between obesity and fitness, the obesity paradox hypothesis has been proposed, which may partly explain our findings [22, 23]. However, considering the positive correlation of obesity and urbanization in modern life [24] as well as the role of obesity in the development of many chronic diseases [16, 25, 26] the influence of obesity on HRQoL should not be ignored, particularly for obesity in young and middle age adults. We did not find that smoking or drinking had a significant influence on HRQoL. This is consistent with the findings by Song et al. [6] among urban community residents in an industrial city located in the northeast of China. Smoking and drinking has been consistently accepted as risk factors for many chronic diseases but are rarely correlated with HRQoL [27, 28]. However, bearing in mind the adverse effects of these behaviors on health and the significant correlation of bad health and poor HRQoL, smoking cessation and alcohol abstinence should continually be advocated as part of public health education. We also did not find any influence of sociodemographic and socioeconomic variables such as education levels, income, insurance, marital status and employment on HRQoL in the urban community population. HRQoL is a multifactorial complex concept, and the interrelationship of many factors can influence it. The findings of our study does not suggest that these sociodemographic and socioeconomic variables are significant determinants for HRQoL. However, further investigation is required to dissect the complex interactions of these variables and other factors.

It is envisaged that age is an important determinant of HRQoL. In our final stepwise regression models age, was negatively associated with physical health and positively with mental health. Thus age-specific health promotion for better HRQoL is necessary. Sleep quality and appetite are two health behaviors that were strongly associated with HRQoL, particularly for mental health. It indicates that sleeping well and good appetite are important for HRQoL. Any health problems that disturb sleep pattern or reduce appetite need to be attended to for a better HRQoL. Daily routines and regular leisure time exercise are two habits that were significantly associated with higher scores of HRQoL for physical and mental health. These positive habits should be extensively promoted at the community level. Currently, the exercise routine of square dancing set to music in squares, parks and any open spaces is popular in Chinese communities [29], particularly for retirees and unemployed residents. This practice is expected to be a good health promotion strategy to increase physical activity and maintain a daily routine, and could be encouraged in the Chinese communities to increase HRQoL. Our data also show that life satisfaction is an important determinant for HRQoL of physical and mental health. To hold an optimistic attitude to manage chronic diseases and to cope well with adverse life events will be of benefit for HRQoL.

## Conclusion

Residents living in newly developed neighborhoods have a lower prevalence of chronic diseases and higher HRQoL scores relative to residents living in the old city central community. Chronic diseases are an important determinant on HRQoL. Several healthy habits and behaviors can be promoted to have a better HRQoL in Chinese urban communities. Targeted policies of public health based on the findings in the present study might improve the health-related quality of life.

### Limitation

There are limitations in the present study. This is a cross-sectional study that only can identify associations. The causality of these associations needs to be investigated by a longitudinal study. In addition, we didn’t require the diagnosis certificate or case of the accident in the course of the investigation, and there is a certain information bias.

## Data Availability

Data is available on request from the corresponding author.

## Abbreviations

HRQoL: Health-related quality of life
CUA: Central urban area
DN: Developing neighborhood
SF-36: The MOS 36-item short form health survey
PF: Physical Function
RP: Role limitations due to Physical problems
BP: Bodily Pain
GH: General Health
VT: Vitality
SF: Social Function
RE: Role limitations due to Emotional problems
MH: Mental Health
PHC: Physical Health Components
MHC: Mental Health Components
COPD: Chronic obstructive pulmonary disease
